# Mr. Lloyd on the Nature and Treatment of Scrophula

**Published:** 1822-06-01

**Authors:** 


*A Treatise on the Nature and Treatment of Scrophula; describing its Connexion with Diseases of the Spine, Joints, Eyes, Glands, &c. founded on an Essay to which the Jacksonian Prize for the Year* 1818, *was adjudged by the Royal College of Surgeons; to which is added, a brief Account of the Ophthalmia, so long prevalent in Christ's Hospital.* By EUSEBIUS ARTHUR LLOYD, Member of the Royal College of Surgeons in London; Senior Surgeon to the General Dispensary, Aldersgate Street; and late House Surgeon to St. Bartholomew's Hospital. Octavo, pp. 342. London, 1821.

HIPPOCRATES, and the primeval physicians of Greece, Italy, and Arabia, invariably recognized in scrophula the distinctive characters of a local disease. Without alteration, this view of its nature continued to glide along the current of medical philosophy, till the close of the sixteenth century, when certain eminent pathologists in England, France, and Germany, began to promulgate doctrines assumptive of its origination from a constitutional source. These doctrines suggested perhaps by a change in the scrophulous phenomena themselves, or by the speculative efforts of genius disentangling itself from the trammels of authority and empiricism, have never ceased to be admitted or modified by those spirits which guided the march of science, from the period intermediate to its revival and that of its ascendancy in our own times.

Prefixed to Mr. Lloyd's work, is a dedication to Mr. Abernethy, in which he declares—

"That one of the principal objects of his book is to establish in a single but important instance, the truth of those principles which Mr. A. has so long maintained concerning the dependence of local diseases on general disorder of the system, and particularly on disorders of the digestive organs, and which have now forced their way through all the opposition of prejudice and old opinion, in a manner which marks at once their inherent excellence and truth."

Mr. Lloyd divides his treatise into two parts. In the first he treats of scrophula in general, under three sectional heads;—of its characteristic signs—of its origin or causes—and of the curative means best adapted to remove that peculiar state of constitution on which it depends. He describes in the second, the topical effects produced by this state of constitution—the particular changes determined by it in different structures—and the local treatment of these structural changes, under the various modifications they are known to assume.

Part. I. *Pathology.* Mr. Lloyd is not unsuccessful in his attempts to establish, by characteristic indications, a distinction between the scrophulous state itself, and the morbid disposition from which originates this disposition, which really exists in nature, and is at all times accessible to observation. It has also been perspicuously drawn in Dr. Thomson's Lectures,* the superlative merits of which will never cease to secure to them a prominent situation in every medical library.

"The term scrophula," says the professor, "is used by medical writers in two senses, first to express the existence of a disease, which seems to possess certain distinctive characters in whatever part of the body it may appear; and, secondly, to indicate a disposition, diathesis, or state which predisposes some part or other of the body to become affected with scrophulous diseases."

This malady, whatever be the form it assumes, is as insidious in its approach as it is gradual in its developement. Various constitutions and very dissimilar textures are susceptible of the morbid actions on which it depends; and these constitutional as well as textural circumstances, in a particular manner, determine its characteristic manifestations. Children and the young suffer oftener from this disease than those of riper years. From its influence, however, no period of life or condition of sex is altogether exempted. Age, indeed, alters the predisposition to it, in different structures. In childhood and adolescence, the upper lip, the eyes, and the glandular system, are prone to become the site of scrophulous lesions: the lungs, the visceral tissues, and spongy part of bones, acquire an increased degree of liability to sustain their ravages in after-life.

*Symptomatology.* Systematic writers, in general, regard the scrophulous habit as being characterised by a great assemblage of distinctive symptoms. Mr. Lloyd's symptomatography of it is less comprehensive. Persons in whom it obtains, according to his observations, are distinguished by a particular delicacy and languor of countenance; their cheeks are soft, smooth, and flaccid; the lips retain a mellow redness, while the parts around the mouth are of a dull pallid hue; there is an indescribable appearance about the

---

* Lectures on Inflammation, p. 132.

eyes; the pupil is considerably dilated; from its vessels being impermeable by the red globules of blood, the conjunctival membrane exhibits a pearly whiteness; and the superior eyelid is unusually depressed.

Mr. Lloyd believes, and universal experience confirms the remark, that there are no legitimate grounds for regarding the white and rosy cheek, the flaxen hair, and azure eye, as symptoms indicative of a predisposition to this disease. He is fully convinced, from very extensive investigation of the subject, that persons having every variety of complexion, from the fairest European tints to the darkest Ethiopian, are alike exposed to it: and that it is only necessary to place them in circumstances favourable to its development, to have it fully established.

*Etiology.* Mr. Lloyd considers the origination of a scrophulous state as being, *in some degree*, determined by the simple or combined operation of such causes as the following:—cold and variable temperature; excessive humidity and impureness of the atmosphere; mental disquietude; inactive, luxurious, intemperate life; indigestible, defective, or insalubrious nourishment; precarious health induced by inflammatory, febrile, and nervous diseases; debility resulting from the action of mercury; contagions of various kinds; hereditary or congenital peculiarity of organization; and, *in a chief degree*, all those agents whose influences derange the vital, particularly the digestive functions; and, by altering the natural actions of a part, give rise to that change of structure which is termed a scrophulous disease.

According to Dr. Thomson of Edinburgh, scrophula "has been observed to occur in children who, instead of being suckled at the breast of the mother, are fed with the spoon; in others, who, though suckled at the breast, have had only a scanty allowance of old and vitiated milk; and also very frequently in those who, while young, could obtain only a watery vegetable aliment."

Among its exciting causes he likewise enumerates the impure air of crowded schools, hospitals, and manufactories; too warm clothing in bed, and two little when exposed to the air; and every thing which can tend, either more directly or more remotely to weaken the general system, or to induce debility.

Illustrative of the suddenness and certainty wherewith derangement of the alimentary functions produces local mischief, Mr. L. adduces five pathographical histories; and by their inductive testimony, regards the doctrine as being satisfactorily confirmed. We transcribe the first.

"A poor man was brought into St. Bartholomew's Hospital with a compound fracture of his leg, produced by the kick of a horse: the tibia protruded, and the parts around were much bruised, and sloughed away to a considerable extent, but the chasm became filled up with healthy granulations, and at the end of a fortnight the man was in particularly good health, free from all fever, or any species of irritation. At this time, which was on a Sunday, his friends, who lived in the country, came to see him, and brought him a large plumb pudding, and other substantial provision, of which he ate most heartily, and in the evening was quite sick. During the night a high degree of fever came on, and towards morning he became delirious. Upon examining the wound to dress it, which was before perfectly healthy, its edges were found in a state of mortification, the granulations were all absorbed, and the tibia was exposed; so remarkable was the change which had been produced in the state of the wound, by this accidental derangement of the digestive organs, in the short space of twelve hours. On the following evening the wound was surrounded with erysipelatous inflammation, which, on the next day, was spreading so rapidly, that the limb was obliged to be amputated: this, however, had no effect in stopping the constitutional disorder, as the man died about eighteen hours after the operation."

Mr. Lloyd coincides in opinion with those who admit that scrophula is, in frequent instances, an hereditary disease. He proceeds to establish this proposition by a series of clinical observations: we submit the outline of two which were treated by Mr. Langstaff, in whose excellent museum the morbid parts are preserved.

"A woman died of consumption in the last month of her pregnancy. Her body was examined after death, as well as that of the fœtus. Her lungs were found full of tubercles, some of which had suppurated and destroyed much of the substance of the lung; in other respects the body was in a healthy state. The lungs of the child were found precisely in the same state as the mother's, being studded with scrophulous tubercles, some of which had suppurated. The rest of the body was in a natural state.

The next case is that of a woman, who died also of consumption, a fortnight after her confinement. The child was still-born. Upon examining her lungs, they were found to be in the same state as in the preceding case, being studded with tubercles, some of which had occasioned abscesses in the substance of the lungs. In other respects her body was free from disease.

The lungs of the child were in the same state, and the kidneys also had scrophulous tubercles in them.

*Constitutional Treatment.* This section of Mr. L's work abounds with judicious practical remarks: it also throws additional light on some of the causes which contribute to originate the disease. The author's therapeutic instructions are simple, but not on that account the less valuable. He contents himself with shewing that, in the prevention and cure of scrophula, our attention must be principally directed to the digestive organs. In the prevention, by avoiding all those causes which tend to disturb their functions; and, in the cure, by tranquillizing irritation, and restoring their healthy actions. For the attainment of these general objects, he institutes an appropriate management of clothing, diet, and medicinal agents.

*Clothing.* Frequent vicissitudes of climate have even been injurious to health; and, in this country, are deemed accessary to the prevalence of scrophulous affections. Mr. Lloyd proposes to counteract their effects on the animal economy, by employing means adapted to preserve the temperature of the body, as equable as possible. For this purpose he very properly advises the use of woollen clothing next the skin, and recommends the practice by two cases which we subjoin.

1st. "A young woman, of very delicate health, who had had for several years the lymphatic glands of her neck diseased and suppurating, the one after the other, and leaving ulcers difficult to heal, which were evidently of a scrophulous nature, was attacked with pain in her chest, and cough, which were very obstinate, and resisted all the usual remedies. After this, when it was believed that her cough was incurable, and that she was in a decline, she was recommended to clothe herself in flannel, which she did. From that time her cough began to get better, and in a few months was quite well, as were all the swellings and ulcers in her neck. No new medical treatment was made use of during this period, and all the medicines she took, were linctuses for her cough, and occasional doses of opening medicine. It is worthy of remark, that till she had recourse to additional clothing, she was particularly susceptible to colds, getting catarrh, sore throat, or inflamed eyes, on every trifling exposure to colder air than usual."

2d. "A little delicate boy, who had strumous affections of the glands of his neck, and symptoms of tuberculated lungs. These symptoms had existed for about two years, and during two seasons he had been at the sea-side; but without receiving any positive benefit. He had also, during this time, made use of all the different specifics which are recommended for scrophula; but never acquiring more than temporary relief from any of the remedies, it was determined to try the effect of warmer clothing than he was accustomed to. He was therefore clothed in flannel, and his general clothing was so proportioned as to prevent the variations in the temperature of the air from having sensible effect on his body; under this treatment it was soon perceived that his health improved very much, and in a few months, he had entirely lost his cough, and the enlargement of the glands of his neck had also disappeared. He has, since this time, which is five years ago, remained perfectly free from the disease, and his general health has been extremely good. His diet during this time has been principally milk, but he is not debarred from eating a little meat once a day. This is the whole of the medical treatment that was adopted, and surgically, nothing was done except keeping a piece of soap plaister to the swellings of the neck."

*Diet.* Stimulating food and drinks of every kind are to be carefully rejected by scrophulous patients. They ought to eat no more at one time than can be easily digested; and their meals, consisting solely of good and nourishing fare, should be taken at regular intervals.

" Nothing," says Mr. Lloyd, "can be worse than living irregularly; perhaps fasting for the whole day, and then eating one immense meal, as the natural consequence of it, must be to excite the whole system to a state of fever, besides the immediate bad effect that it has on the stomach and bowels. All stimulating liquors too should be avoided; and, as hunger and thirst are incompatible sensations, eating and drinking should not be indulged in at the same time, but the same regularity should be observed in the one as in the other."

These observations, it should be recollected, apply only to the simple state of a scrophulous constitution, which is not suffering from local disease. In complicated cases, wherein the local disease is extensive and producing irritation, pain, disturbance of the natural functions, and hectic fever, experience has taught us that generous diet and stimulating drinks, are not only admissible, but useful.

*Medicinal Agents.* Consistently with his general views of the disease's nature, Mr. Lloyd estimates the keeping the bowels regular, and the hepatic secretion natural, as the most important point in the treatment. But it will be in vain, he emphatically adds, to attempt to improve the state of the health, or to regulate the action of the bowels by medicine, if attention to diet be not, at the same time, observed. For, says he, if the stomach be overloaded with food, no proper digestion can take place; and, if the food be of an improper quality, or in improper quantity, no medicine can act beneficially on the bowels.

When his patient is an adult, and his bowels obstinately confined, Mr. L. gives five grains of the blue pill every night, with half a pint of the compound decoction of sarsaparilla twice in the day, and if, by a certain hour, the bowels continue unmoved, he superadds some opening medicine, and repeats it at proper intervals till moderate evacuation be produced. He pursues this plan till the alvine functions become regular, and then to prevent their relapsing into the same state, he goes on exhibiting the compound calomel pill in alterative doses of five grains, every second night, for an indefinite time.

Similar principles guide his practice in the treatment of scrophulous children. His observations on this branch of the subject are so judicious, that we shall offer no apology for transplacing them to our pages in the author's own words.

"But," he proceeds to say, "as the constitution as well as the age of children who may become affected with this disease, may be very different, it is impossible to know, at once, what medicine will be applicable to each particular case. Indeed, every one must have observed, that the same medicine may act very differently on children of even the same age: and, that what purges one violently, will have no effect on another. We should too, be very careful not to give violent purges, and we should particularly avoid large purgative doses of calomel, as I am convinced they often produce more general irritation than the evacuation they occasion from the bowels is able to relieve: and that they often so much weaken the stomach, that it is a very long time before it is able to recover its natural powers. Our object, therefore, in prescribing medicines, should be to procure a proper emptying of the bowels daily, and a healthy condition of the secretions. This, I admit, is often a difficult point to obtain, but by proper management, we may generally succeed. Any of the mild purgative medicines may be employed for this purpose, and if one does not appear to have the proper effect, we should desist from its use and substitute another; but we may derive the greatest assistance from exhibiting alterative doses of calomel at the same time. The dose should be varied from half to one grain, according to the age and habit of the child, and repeated twice or thrice a week. Sometimes, in particular states of the stomach and bowels, it is better to combine the calomel with the purgative, and at other times, they act better given separately. The very great influence which evacuations from the bowels have over the rest of the body, cannot be denied by any impartial observers; it is, therefore, certain, that by increasing or diminishing them, we are able to produce a decided effect on the whole, or, as I have proved before, on a particular part of the body. Thus, if there is much general irritation, or local irritation and inflammation, by increasing the intestinal evacuation, taking care, however, not to irritate the bowels, we may very much relieve both the one and the other."

When gastric acidity prevails, he employs soda, preferably to the other alkalis, in small proportions "till the cause of this morbid secretion is removed." In cases of weak stomach, with loss of appetite, he has found cinchona, steel, the mineral acids, and other tonics, to be serviceable, though he is perfectly satisfied that they possess no specific power over the disease. When there is only a disposition to scrophula, and before the supervention of any active visceral disease, he admits that cold sea-bathing may, under certain circumstances, be made beneficial by rendering the body less susceptible of cold, and, therefore, less likely to be influenced by the vicissitudes of climate. Nevertheless, he reckons it altogether incapable of promoting the discussion of scrophulous tumours, or healing the ulcers which proceed from them. He denounces it, moreover, in all cases wherein there is reason to suspect even a disposition to tubercles of the lungs. Instructed by observation and reflection, he contends that, scrophulous patients should, on no account whatever, remain longer than four, or at most, five months in the year at the sea-side. We should be inclined to abbreviate even this period; at least, to divide it by a temporary change of residence.

Mr. Lloyd's experience leads him to reject warm bathing in scrophulous complaints: it hurries respiration, quickens the pulse, and seems to injure the general health. Good air and exercise are conducive to the cure of this disease, by their tendency to promote the regular performance of the vital functions.—From the foregoing observations, of which we have endeavoured to exhibit a concise view, Mr. L. thinks the following conclusions may be fairly drawn.

1st. That scrophula is hereditary, but that the tendency to it may exist without its being called into action. 2dly. That a disposition to it may be acquired where there is no hereditary tendency. 3dly. That all those causes which tend to derange the natural actions of the body, are capable of inducing scrophula, and thus it is always a constitutional disease. 4thly. That the disease may be generally prevented by avoiding all those causes which have a direct influence in disturbing the general health. 5thly. That the disease is only to be cured by avoiding all sources of irritation, and by restoring the natural and healthy functions of the digestive organs.

Part II. When the scrophulous constitution becomes established, there is no structure nor organ of the body which may not be attacked by it, and experience teaches us, that some parts are more obnoxious to its influences than others. In the second division of his Treatise, Mr. Lloyd proposes to illustrate and confirm this doctrine, by directing our attention to the pathology and treatment of local scrophulous disease as it appears in the lymphatic and other glands,—In the female breast,—the testicle,—prostate gland,—bones,—joints,—hip-joint,—spine,—lungs,—heart,—liver,—pancreas,—spleen,—intestines,—kidneys,—brain,—and eyes. We proceed to exhibit a summary view of the manner in which these important subjects are discussed.

GLANDS.—*Pathology.* In a scrophulous gland, at an early stage of the affection, the permeability of its vessels remains; it is simply enlarged from thickening of its cellular structure. As the disease advances, the organ appears to have more nutrient arteries and to be redder than is natural. Gradually, it becomes wholly altered; new matter is deposited, and this sometimes has a firm consistence, resembles cheese, and when divided, shows an even, mottled, yellowish white surface. At other times, it is converted into a substance which is less firm in texture, and when incised, appears to be composed of two kinds of matter, one of these resembles curd, the other is softer, less opaque, and yellow. Minute quantities of pus are occasionally deposited in the centre of such a gland; and, sometimes, when its size is great, it contains small abscesses filled with scrophulous matter, which subsequently makes its way to the surface and gives rise to a suppurating tumour. Inflammation now seizes the integuments, and spreads to the surrounding parts: these go on to suppurate, and together with the newly formed morbid substance, produces a large abscess, by which the gland is totally destroyed. Such is the more common progress of scrophulous inflammation in a lymphatic gland. Instances of its pursuing a different course are, however, not unfrequent: the subsequent remarks of Mr. Lloyd are worthy being transcribed.

"It sometimes happens," says he, p. 53, "that the suppuration only takes place round this newly formed matter of the gland, which may be seen through the aperture through which the contents of the abscess have been discharged, and between it and the sides of the abscess, you may readily pass a probe on every side. When this is the case, the healing of the wound is very tedious; but, that this is the altered substance of the gland, and not sloughing cellular substance, as has been stated, I entertain not the slightest doubt, from having examined it after it has come away in the poultice, subsequently to my enlarging the aperture, either with a knife or with caustic. This complete change in the substance of the gland does not necessarily take place: for it frequently occurs that, the gland being simply enlarged, as mentioned at first, the surrounding parts become affected with scrophulous inflammation, and matter forms, which is at length discharged through one or more ulcerated openings."

Many times, several glands experience simultaneous tumefaction, and continue distinct till they have acquired a very great size. At length, they coalesce and form an immense tumour, which compresses the side of the larynx and pharynx, so as to impede respiration and deglutition. By the circumstances of two appropriate cases, Mr. Lloyd demonstrates the coalescence of glands, and the consequences that may ensue from their excessive enlargment. He then goes on to observe, that not only the lymphatic glands of the neck are liable to undergo scrophulous disorganization, but by a similar process, abscesses have been formed on the middle of the tibia, behind the inner condyle of the femur, above the inner condyle of the humerus, on the inside of the ulna near the olecranon, above the middle of that bone, above the clavicle, in the thoracic duct, in the thymus, thyroid, parotid, salivary and inguinal glands. Sometimes he has found the cervical glands so enlarged as to form a chain passing under the chin from ear to ear, in the form of a horse's shoe. Scrophulous abscesses, moreover, may have place in every part of the body, independently of the glands, as in the chest and angles of the ribs; but, when this is the case, their suppuration is more rapid.

Whenever the thymus gland begins to enlarge from scrophulous tumefaction, it occasions most serious uneasiness.

"On the front," says Mr. Allan Burns,* "the tumour is prevented by the sternum from protruding outwardly: above the sternum, the fascia and muscles repress its growth: as it enlarges, therefore, it must press backwards on the important parts which are between it and the spine. No wonder, then, that the patient should in the end, die from suffocation and starvation. Even what food passes into the stomach, fails to nourish the body properly. The pressure of the tumour on the subclavian vein, interrupts the entrance of the chyle into the heart, and thence, the mesenteric glands are, in such cases, generally found enlarged and obstructed. In three children who had died from disease of the thymus gland, I found the lacteal glands increased in size."

When topical and internal remedies have failed of curing or mitigating the disease,

"It is practicable," adds this consummate anatomist, "to excide this tumour, and I have done it twice on the dead subject. To do this, I made an incision on the front of the neck, just above the sternum, and between the sterno-hyoid muscles, as in the operation of tracheotomy. By this cut, the rounded knob of the diseased thymus was exposed. Having done this, I next insinuated the forefinger between the gland and the adjacent parts, till the former was

---

* Surgical Anatomy of the Neck and Head, p. 9.

insulated so far as I could reach. After this, by a pair of polypi forceps, cautiously introduced between the mediastinum and the gland, I grasped the tumour, and wrenched it from its connexions. This, on the living snbject, would be a most dangerous operation, yet, where death is otherwise inevitable, it might perhaps be warrantable to try it. I think, that were it cautiously executed, injury of the large vessels might be avoided, and the sponge would easily command any bleeding which might take place from its own nutrient arteries: an event which is hardly possible if the tumour be pulled away. Some may suppose, that inflammation would be apt to follow this operation, but this is to be little dreaded: the debilitated state of the patient will be a sufficient security against its occurrence."

There are few histories of the thyroid gland having suffered scrophulous disorganization, on record. It is much more frequently the seat of goitrous tumefaction. By Fodéré* the discriminative symptoms which distinguish scrophula from bronchocele, have been perspicuously and eloquently stated in his interesting Treatise on the Nature and Treatment of the Thyroideal Tumour. Portal† regards the parotid and all the salivary glands as being exposed to scrophulous engorgement. Mr. Allan Burns,‡ however, is of opinion that the parotid itself is not so often the seat of morbid enlargement, as the conglobate glands by which it is surrounded. He has taken considerable pains to establish the impracticability of extirpating this organ, and thinks that, those authors who have described their performance of the operation, had done no more than merely excide one of the glands, situated external to the parotid. His reasonings in confirmation of this doctrine are so ingenious, and apparently so satisfactory, that we should have found ourselves induced to admit the same conclusions, had we not been aware that this would be, not only to discredit the anatomical knowledge, but to affirm the positive ignorance or falsehood of others, which, we conceive, it would be more decorous to demonstrate, than to assume.—We can refer our readers to six authentic details of the operation; and, we believe, the first four of these have hitherto been unknown to many individuals in this country. The parotid gland, then, was excided by Burggrav,§ in 1727; by Hetzel,|| in 1767; by Siebold,¶ in 1780; by

---

* F. E. Fodéré.—Traite du Goitre et du Cretinisme, 1800.

† A. Portal.—Cours d'Anatomie Médicale, Tom. II. p. 9.

‡ Allan Burns on the Surgical Anatomy of the Head and Neck, p. 271.

§ Johannes Phillipus Burggrav.—Parotis Scirrhosa multum protuberans feliciter resecta; in Act: Academ: Natur: Curiosorum, Vol. I. 1727.

|| David Franciscus Hetzel.—Extirpatio Glandulæ Parotidis Scirrhosæ; in Nov: Act: Academ: Natur: Curiosorum, Vol. III. 1767.

¶ Carolus Caspanus Siebold.—Parotidis Scirrhosæ feliciter exstirpatæ Historia; in Act: Academiæ Moguntinæ, 1781.

Lacoste,* in 1806, by Palmer† in 1812, and by Carmichael‡ in 1817; and till the inadvertency, the nescience, or the mendacity of these men shall be proved, we must admit the practicability of accomplishing extirpations of this intricately situated organ.

*Treatment.* Considerable importance is attached by Mr. Lloyd to the treatment of scrophulous tumors. We copy his own description of the process by which he endeavours to promote the resolution.

"When," says he, p. 63, "the glands have become simply enlarged, and appear in an indolent state, the less that is done to them the better, as nothing but constitutional remedies appear to have much influence upon them in that state. It may, however, be right to bathe the part with salt and water, or any other cooling wash, to prevent the surrounding parts becoming irritated by the pressure made on them by the enlarged gland. In the more advanced stages of the disease, the treatment consists in allaying irritation by soothing applications, and by applying leeches if there is much pain. When several enlarged glands, having remained in an indolent state for some time, suddenly begin to enlarge, are painful to the touch, and seem disposed to coalesce, although the superincumbent skin is neither discoloured nor tense, the application of leeches and cooling washes is often highly serviceable: but when they have coalesced, forming a large tumour, and the skin above is tense and discoloured, the best applications are warm emollient poultices, as they tend to take off tension, and consequently allay irritation. If, under these circumstances, it be judged necessary to apply leeches, on account of the great irritation and pain, they should not be applied to the discoloured skin covering the tumours, but rather at some little distance from it, and they may be serviceable though without this precaution they might have increased instead of diminished irritation. It often happens, that when the swellings have arrived at this height an abscess forms: but it also happens that they become indolent, and the pain and tension both subside. The tumour, however, remaining undiminished, will, upon examination, be found to contain, in its upper surface, a small quantity of fluid. In this case the application of a blister, to be kept open for a few days, and repeated according to circumstances, will often promote rapid dispersion of the fluid, and indeed sometimes of the whole tumour. The appli-

* Barthelemy Lacoste.—Observation sur l'extirpation d'un glande parotide squirreuse—Rec. period. de la Soc. de Med. de Paris, tome 26th, 1806.

† Dr. Palmer, of Tamworth.—Med. Chir. Journal, vol. 1st, 1816; and Med. Chir. Rev. Vol. ii. 1819.

‡ Mr. Carmichael of Dublin.—Transac. of Irish Col. of Physicians, Vol. ii. 1819.

cation of blisters and other stimuli in glandular swellings, without great discrimination, would, I believe, be attended with much mischief."

*Scrophulous Ulcers.* The only characteristics of a scrophulous ulcer that can be depended upon, according to Mr. Lloyd, are—its occurring after a scrophulous abscess; the peculiar dull red or purple colour of its edges: its remaining indolent for a great length of time, neither increasing nor diminishing in size, and its being attended by that particular state of health which invariably prevails in the scrophulous constitution. His treatment of such ulcers consists in "attention to the patient's diet, and to his stomach and bowels, and in soothing applications to the local disease." Several rather interesting cases are adduced in illustration of the advantages which this practice combines: of these we select one.

"A boy, six years old, of fair complexion, blue eyes, and light hair, was in Sept. 1816, brought to me with abscesses on various parts of his body; he had a bad cough and difficult respiration, tumid belly, and pallid countenance; his bowels were too open, and had been for some time irregular, and what was discharged from them was very offensive and dark coloured; but his appetite was very good. He had every night a regular attack of hectic. As the bowels were too open, and the secretions so bad, being almost black, four grains of the hydrargyrus cum cretâ were given him every night, and continued for the first fortnight: but afterwards only every other night. He was put on milk diet, being allowed a pint a day, and in every respect he was treated upon the principles which I have laid down. It was astonishing to witness how much he improved during the first six weeks under this treatment: but after this the bowels again became disordered, and the hectic and all the bad symptoms returned. He lingered in this state, getting more and more reduced, for about two months, and then died.

"Upon examining his body, the lungs were found very much diseased, and full of tubercles; and the mesenteric glands were also very much diseased. Some of them were in a state of suppuration, no vestige of the gland remaining; others were converted into a cheesy sort of substance; some were much enlarged, with a deposition of soft cheesy matter in their centre, while others were simply enlarged."

*Mammary Scrophula.* Scrophulous affection of the female breast seems to be modified to a certain extent, by the patient's age. In young women, it generally commences with the formation of a hard moveable tumour in some part of the organ. This tumour progressively increases in size, and coalesces with the surrounding parts which become tender, inflame, suppurate and break into small ulcerative openings, that are sometimes very difficult to heal. Occasionally also, the whole skin of the lower portion of the breast, where the abscesses are generally seated, becomes discoloured and diseased, the tumefaction augments, and a considerable degree of constitutional irritation ensues, which is seldom much relieved by general or local bleeding. Under such circumstances, Mr. Lloyd advises the insertion of a seton, and exhibits its beneficial effects in the following case.

"A young woman, aged twenty, had a small hard lump form in the under part of the breast, which gradually enlarged till it was lost in the surrounding parts; which became painful, inflamed, and formed an abscess, which discharged about three ounces of a scrophulous matter. A small ulcer was left; and the parts which were covered with a bread and water poultice became in a quiet state. Her health had been for many months in an indifferent state, and her catamenia were suppressed. The constitutional treatment that was adopted consisted principally in keeping her bowels open, and in attention to her diet.

"She, however, did not materially improve in health; and abscesses continued to form successively: the swelling of the breast rather increased, and was attended with great pain. I therefore took from her arm twelve ounces of blood, and for the first two days the pain was less, and her pulse more tranquil; but the pain again returning, I bled her again to the same extent but without benefit. Leeches were also applied repeatedly to her breast, but with the same success. She now became very impatient: I therefore made a seton under the breast, at a little distance from it, and continued the treatment that had been previously adopted. From this period, a complete change appeared to take place in her, as both her health and her breast began rapidly to amend; so that in less than two months the breast was reduced to its natural size; her catamenia re-appeared, and her health was very good: and in less than another month I removed the seton, as the ulcer had healed, and only a slight hardness remained."

Mammary scrophula, in the adult age, is generally distinguished by enlargement of all the glandular structure of the breast, which becomes preternaturally firm, while the skin and subcutaneous textures continue soft and uncoherent with the condensed parts. This state, unattended with pain or even tenderness on pressure, will sometimes continue for many months, or even years, or the disease's progress will be so gradual as to be almost imperceptible, till suddenly the sound textures sustain inflammatory excitement, and are consolidated with the morbid gland. The tumour now becomes very painful; great constitutional disturbance arises; partial suppuration ensues; a small superficial abscess forms, bursts, and, through a minute orifice, discharges an inconsiderable proportion of curdy whey-like matter. After an indefinite time, the opening will cease to discharge, and cicatrize: successive abscesses will in the same manner be formed; be attended by the same symptoms, and pursue the same course, till the tumour is progressively dissipated, and the whole breast wasted away.

"During the formation of these abscesses," says Mr. Lloyd, p. 87, "if we trace the surface of the breast with our finger, it often appears modulated, and particularly at those parts where the superincumbent integuments have not become firmly attached to the subjacent gland; but sometimes it appears as if there are several soft places or pits, in the surface of the tumour, into which we can readily introduce the top of the finger. It is also very common, during the progress of the disease, for abscesses to form in the course of the absorbents passing to the axilla, and in the glands of the axilla itself, and even before any abscess has formed in the breast; but these are of no consequence, and always cease with the original disease."

Regulation of the patient's diet and alvine functions are here indicated: and when the constitutional disturbance is great, with a weak rapid pulse, occasional exhibitions of some neutral salt, with small doses of hyoscyamus, will be found very useful. At the same time, the application of poultices to the part is indispensable, and the decoctions of poppies and hemlock are useful adjuvants. Leeches may sometimes be employed, but their beneficial effects are uncertain. An issue, however, may be formed by enlarging with caustic the aperture through which the matter has been discharged. It prevents the formation of sinuses, expedites resolution of the tumour, and softens the remaining induration of parts.

This section is finished with some inconclusive remarks on the similarity of appearance exhibited by the scrophulous and scirrhous affection of the female breast. Mr. Lloyd states his belief of the former having been mistaken for the latter, by surgeons who are not conversant with it, and asserts that he has seen the organ "amputated for this disease, upon the supposition that it was cancer." We wish not to palliate the errors of inattentive or ignorant operators, yet we are most certain that many cases, at all times, exist, in which there would be more science as well as humanity in exciding the morbid parts at once, than in leaving them to be destroyed by the tedious, painful, exhausting, and uncertain process of ulcerative inflammation, by which nature struggles to regenerate their health.

Testicular Scrophula—*Pathology*. Children and the young are exposed to suffer scrophulous disorganization of the testicle. One case only of this kind has occurred to Mr. Lloyd, and the disease had made considerable progress before it came under his observation. When the child, three years and a half old, was brought to him, the scrotum was distended with matter, appeared like a scrophulous abscess in any other part, and the skin was transparent. Poultices were applied, and in a few days the integuments burst, the aperture increased, and more than half of the gland projected through the opening. It was converted into a mass of yellow scrophulous matter, which separated soon afterwards, leaving the remainder of the organ enlarged and hardened. This, at the time of Mr. L's writing, "was rapidly diminishing, and seemed likely to entirely waste away."

When the testicle in an adult, is first attacked with scrophula, it gradually enlarges and becomes softer than it is in health. It preserves its natural form, however, and seldom attains a very great size. This stage of the disease is not usually attended with much pain, or even tenderness, on pressure; and its progress is not dissimilar from that of the same affection in an absorbent gland.

The morbid substance which constitutes this enlargement is of two kinds. In the first it is a parenchymatous matter contained in a cyst, and arises from interstitial deposition, or simple expansion of the glandular structure. In this state a small abscess forms in the substance of the testicle, and may exist for an indefinite time without inducing apparent alteration in the membranes or surface of the organ. In the second, the natural structure of the gland comes to be entirely absorbed; and a cheesy, yellowish-white or greenish yellow substance, resembling what is found in the cancelli of scrophulous bones, is deposited in its place. The coats of the testicle, however, continue for some time healthy and unaltered. The morbid deposition occasionally consists of several distinct masses, each of which is invested with a cyst.

So long as the coats of the testicle remain in their natural state, the pain, in either form of the disease, is not very severe: in the latter, however, it is greatest. When the tunics, the scrotum, and other parts inflame, and suppuration commences, the pain is sometimes excruciating; the testicle and scrotum become consolidated, and, when the enlargement is of the first kind, it feels hard, and is not very tender to the touch. In the second kind, the parts are softer and more irritable: and when small abscesses are in progress, if we trace the surface of the gland, we find soft places or pits with hardened edges, into which the point of the finger can be pressed. This state precedes the bursting of these abscesses, through the apertures of which a probe will readily pass into the substance of the gland. Sometimes the matter will be absorbed, and the testicle reduced to its original size, or keep dwindling away till the whole gland is destroyed.

*Treatment.* The first stage requires suspension of the testicle in an appropriate bandage, and the application of bread poultices or cooling washes. Moderate leechings are useful in the second; and the parts should be constantly covered with bread and water poultices. When there is an increase of pain and disturbance of the general health, attended by a weak hurried pulse, sedatives, combined with mild purgatives, produce very beneficial effects. Mr. Lloyd, by the following very impressive case, is solicitous of directing the attention, in a particular manner, to the re-establishment of constitutional health.

"A young man, aged twenty-three, had disease in his left testicle, which was about three times its natural size. It had been gradually enlarging for three months before his admission into the hospital, but during this time there was but little pain in it: when, however, he came into the hospital, it was more painful and tender to the touch, and he complained of pain in his loins. He was very much out of health, and had a weak and rapid pulse. The scrotum now became inflamed, and attached to the outer surface of the gland: and matter formed at this point. At this period leeches, poultices, and fomentations, were had recourse to, but without producing any effect: and as the tension and pain were great, a puncture was made at the most prominent point of swelling, and a desert spoonful of a sero-purulent matter discharged. This afforded some relief; but as the enlargement did not subside, as there was still pain, and the wound continued discharging, a seton was inserted at the upper part of the scrotum. This, however, not only did no good, but produced great swelling and inflammation of the whole scrotum, so that it was obliged to be taken out after it had been in for above a month. All the various means that have been recommended for discussion of enlargements of the testicles were now tried, without any good effect: and as the health of the patient was getting worse, he left the hospital, and went into the country. A few months after this, he called on me with his health much improved; and the testicle was almost of its natural size, entirely free from pain, and the wound perfectly healed. The only remedies he made use of were bread and water poultices, and medicines to keep his bowels regular. Not long after this, however, the gland again swelled, but soon subsided, by the use of the same means; and since that he has remained well a space of nearly three years. I saw him lately in good health; and he now gains his living by driving a hackney coach."

When, in the third stages, the testicle has been partially or totally converted into a mass of caseous matter, which has come away by an external opening; or, when abscesses have formed in the body of the gland, discharged themselves externally, and produced an unhealthy state of the scrotal integuments, with sinuses difficult to heal; the principal object certainly is, to cure the local disease as expeditiously as possible. For this purpose the sinuous orifices are to be enlarged, and the morbid part of the scrotum destroyed by successive applications of the caustic potash, when the sinuses and wounds will soon cicatrize, and the glandular enlargement rapidly subside.

Every surgeon conversant with the history of the science knows that Mr. Percival Pott considered all cases of chronic fleshy enlargement of the testicle, which are attended by a bad state of health, or do not readily yield to the discutient remedies usually employed, as requiring early castration. Notwithstanding the high authority of this celebrated writer, Mr. Lloyd, with becoming modesty, expresses his dissent from the opinion. He unfolds his views of the disease's nature; defines the object of his practice; adduces his pathological facts; and with decent firmness advances the new doctrine. It is long since we ourselves taught that whatever is the effect of lymphatic exhalation, whether healthy or morbid, may be the subject of lymphatic absorption: and we find great satisfaction in admitting the sentiments of Mr. Lloyd, who, although he had done nothing else than establish the possibility of reducing the sarcocelic testicle, would have deserved well of the profession and of mankind.

*Prostatic Scrophula.* On cutting into the prostate, Dr. Baillie has found it containing the very same white curdly matter which is peculiar to a scrophulous absorbent gland. Mr. Lloyd has not met with any instance of this kind; but several cases of sarcomatous enlargement of the gland, in which were small abscesses filled with perfect scrophulous matter have come under his observation. The following possesses considerable interest.

"A young man, aged nineteen, was brought into the hospital with retention of urine. At this time a catheter was attempted to be passed, but without success: and after general and local bleeding had been employed, the attempt was repeated, but with no better success. As, however, the urine was now continually dribbling away, though, I may observe, this produced no diminution of the tumescence of the bladder, and the pain was not so very great, the usual remedies for taking off spasm and irritation were employed; but during their trial the bladder suddenly gave way, and the urine was extravasated into the cavity of the abdomen; and a few hours after the patient died. Upon examination after death, the prostate gland was found of the size of a child's head, but its substance was very much of its natural density. A tumour had formed on its under part, and projected into the cavity of the bladder. It pressed so much on the sacrum, that there was scarcely any room for the passage of the fæces. It had enlarged unequally at the sides of the urethra, by which, as well as by the tumour at its inferior part, obstruction was occasioned to the introduction of a catheter into the bladder, and to the direct transit of the urine. There was a false passage through the under portion of the prostate. In this case the mesenteric and the lumbar glands were enlarged; and one of the latter was converted into a mass of complete scrophulous matter. The bladder was thickened and highly inflamed, and almost in a state of gangrene; and a portion, of about the size of a shilling, at its upper part, had sloughed away, through which the urine had been extravasated. The death was produced by an excessive degree of peritoneal inflammation, which came on immediately after the extravasation of the urine."

This affection of the prostate gland sometimes occurs, though under a less severe form, in young men of a scrophulous habit. It is generally attended with a great deal of irritation about the urethra and neck of the bladder, and by gleet, which is much increased by sexual intercourse. It commonly gets well by tranquillizing the constitutional disturbance, making use of mild local applications, and occasionally introducing a bougie, for the purpose of removing the frequent desire of voiding urine, which so often attends this complaint.

When scrophulous abscesses form in different parts of the prostate, the matter sometimes makes its way into the bladder, near the urethral origin. Sinuses are thus left in the substance of the gland; and through these the urine insinuates itself into the cellular texture, around the anus and perineum. This accounts for the successive production of urinary abscesses, when there is a pervious state of the urethra, and they have not been preceded by retention of urine. Their treatment involves a two-fold object—immediate relief, and an ultimate cure. The former requires puncture of the bladder; a judicious use of the bougie will fulfil the latter intention.*

* The important divisions of our author's work, embracing *osseous*, *articular*, and *visceral* scrophula, we must reserve for our next number. Our readers are aware that we rarely divide the analysis of a work into two articles—and *never* unless there be such natural or artificial divisions in the work itself as will permit this separation without injury or inconvenience to author or reader. Under these conditions we take leave to stop here, for the present; and shall finish with a comprehensive review of the remaining subjects in our next.

